# Flagellin concentrations in expectorations from cystic fibrosis patients

**DOI:** 10.1186/1471-2466-14-100

**Published:** 2014-06-09

**Authors:** Viviane Balloy, Guiti Thévenot, Thierry Bienvenu, Philippe Morand, Harriet Corvol, Annick Clement, Reuben Ramphal, Dominique Hubert, Michel Chignard

**Affiliations:** 1 Unité de Défense innée et Inflammation, Institut Pasteur, Paris, France; 2 Défense innée et Inflammation, Inserm U874, Institut Pasteur, 25, rue du Dr Roux, Paris 75015, France; 3 Université Paris Descartes, Sorbonne Paris Cité, Faculté de Médecine, UPRES EA 2511, Paris, France; 4 Groupe Hospitalier Cochin – Broca – Hôtel Dieu, AP-HP, Laboratoire de Biochimie et Génétique Moléculaire, Paris, France; 5 Université Paris Descartes, Sorbonne Paris Cité, Inserm U1016, CNRS (UMR 8104), Paris, France; 6 Hôpital Cochin, AP-HP, Service de Bactériologie, Paris, France; 7 Hôpital Armand Trousseau, AP-HP, Pneumologie Pédiatrique, Paris, France; 8 UPMC, Université Paris 6, Inserm, UMR-S U938, Paris, France; 9 Université François Rabelais, UMR 1100, Tours, France; 10 INSERM, UMR 1100/EA6305, Tours, France; 11 Hôpital Cochin, AP-HP, Service de Pneumologie, CRCM adultes, Paris, France

**Keywords:** Sputum, Chronic inflammation, Infection, Respiratory function

## Abstract

**Background:**

The aim was to measure flagellin concentrations in the expectorations of CF patients and to examine whether there are correlations with the level of respiratory insufficiency and inflammation.

**Methods:**

Sputum samples from 31 adult patients chronically colonized with *P. aeruginosa* were collected and analysed for their content of flagellin and IL-8. Clinical data were extracted from patient files.

**Results:**

Regardless of whether patients are colonized with mucoid strains or not, they carry clones of *P. aeruginosa* that express flagellin. While flagellin was present in airways of all of our CF patients, it is difficult to ascertain its contribution to inflammation (IL-8) and lung function deterioration.

**Conclusions:**

This is the first demonstration that flagellin is present in the sputum of patients. Thus, attempts to down regulate inflammation by the use of TLR5 (flagellin receptor) antagonists remain a possibility. However, this result needs to be extended to a larger number of patients to validate it for future research on this subject.

## Background

Cystic fibrosis (CF) is characterized at the lung level by a florid chronic inflammatory response which is due to many factors, including proteases such as neutrophil elastase but also chronic respiratory infection, involving most frequently *Pseudomonas aeruginosa* (*Pa*) in adult patients [1,2]. Pulmonary exacerbations occur, possibly due to changes in the metabolism or phenotype of *Pa*[3,4], and contribute to deterioration of the patient’s respiratory status. Of note, the effect of gender and the menstrual cycle has been shown to affect *Pa* phenotypes [5]. Thus chronic colonization of the lungs with *Pa* is considered to be the leading cause of respiratory morbidity and mortality in CF patients [6,7].

The inflammatory response to *Pa* is believed to be mediated mainly by Toll-like receptors (TLR) 4 and 5 [8-10], and possibly by TLR2 [11]. Thus it may be hypothesized that both lipopolysaccharides (LPS) and flagellin expressed and released by *Pa* play crucial roles in lung inflammation since TLR4 and 5, are both expressed by alveolar macrophages, neutrophils and respiratory epithelial cells [11-14]. Some reports seem to indicate that flagellin is possibly an important agonist to initiate an inflammatory reaction as LPS is believed to be poorly recognized by human respiratory airway cells [15,16]. Nevertheless, in acute lung infections of mice, the recognition of LPS by the airways appears to be effective in generating a protective inflammatory response [10], therefore one cannot exclude a role for LPS in inducing the chronic inflammatory response. Indeed, proinflammatory lipid A alterations have been reported in *Pa* strains of CF patients [17].

In contrast to lipid A, the signaling part of the LPS molecule, whose expression is essential for bacteria viability, the expression of flagellin by *Pa* colonizing the lungs may fluctuate and non flagellated strains are frequently isolated from CF patients [18]. For example, *Pa* growing in mucopurulent human respiratory mucus from CF patients represses the expression of its flagellin [19], in response to the presence of neutrophil elastase in such mucus [20]. It is hypothesized that these changes are an attempt by the bacterium to minimize the innate immune response against it. Furthermore neutrophil elastase is also able to directly cleave flagellin, resulting in the loss of its ability to induce an innate host response [21]. In addition, it is thought that *Pa* within a microcolony or biofilm-growing mucoid (alginate-producing strains) possess a unique phenotype quite different from their planktonic counterparts [22,23], repressing genes for flagellar biogenesis in addition to others [24,25]. Hence one can easily conclude that flagellin may or may not be expressed depending on the environment, and thus may or may not play a role in the chronic inflammatory response.

The aim of the present study was thus to measure flagellin concentrations in the expectorations of CF patients and to examine whether correlations exist with the presence or absence of flagellin and the level of respiratory insufficiency. This is an important question as in case of the presence and of a positive correlation one can envisage anti-inflammatory treatment based on the inhibition of the interaction between flagellin and TLR5 by mutants of flagellin or anti-TLR5 small molecules. Indeed, it has been already shown with human respiratory epithelial cells that a mutant of flagellin that activates TLR5 poorly, was able to reduce IL-8 synthesis triggered by wild-type flagellin [26].

## Methods

### Patients

Thirty-one adults with CF attending the CF centre at Cochin University Hospital, Paris were enrolled between May 2011 and October 2011 during a planned visit. None of the patients was hospitalized. Criteria for inclusion were: age older than 18 and chronic colonization with *Pa* (defined according to the Lee criteria [27] when airway samples were *Pa* culture positive in > 50% of the explored months over the last 12 months). Clinical data were extracted from patient files, including demographic data (age and gender), CFTR genotype, exocrine pancreatic insufficiency, diabetes, body mass index and pulmonary function. Pulmonary function tests were performed on the day of the outpatient visit. Forced expiratory volume in one second (FEV1) and forced vital capacity (FVC) were expressed as percentages of the predicted value (% pred.). Patients were considered stable or in exacerbation according to Fuchs criteria [28]. They gave their informed consent for participation in the study which was conducted in accordance with the Declaration of Helsinki and French law and was approved by the Institutional Review Board for Medical Research (CCTIRS # 08–370). In addition, patients provided consent for the publication of their data.

### Sputum collection and treatment

All sputa were spontaneously expectorated and were collected in a sterile container. A fraction of the each sputum sample was processed by the Clinical Microbiology Laboratory of the hospital for microbiological analysis. *Pa* was quantified by platting serial dilutions (1/2, 1E-3, 1E-5) of fluidified (Digest’Eur, Eurobio, Courtaboeuf, France) sample on non-selective (COH and PVX, BioMerieux, Marcy L’Etoile, France) and selective (Dirgalski, BioRad, Marnes La Coquette, France) solid media. Plates were incubated for up to 7 days. Quantification and mucoid phenotype (absence/presence of viscous and slimy colonies) of each *Pa* isolate was reported upon bacterial isolation. The other fraction of the sputum was left on ice and processed within two hours from the start of the collection. All material was transferred to a Petri dish to discard saliva, then the sputum was collected and vortexed for 1 min with v/v RIPA lysis buffer 2× (300 mM NaCl, 50 mM HEPES, 10 mM EDTA, 0.2% SDS, 2% Nonidet P40, 0.1% Deoxycholate), supplemented with Complete, EDTA free-protease inhibitor cocktail (Roche Diagnostics). Samples were centrifuged and the supernatant was stored at −80°C.

### Flagellin quantification

Flagellin amounts in the expectorations were quantified by Western Blot analysis by comparing the band intensity to a standard curve of purified Pseudomonas flagellin, purified as described previously [26].

**Figure 1:**
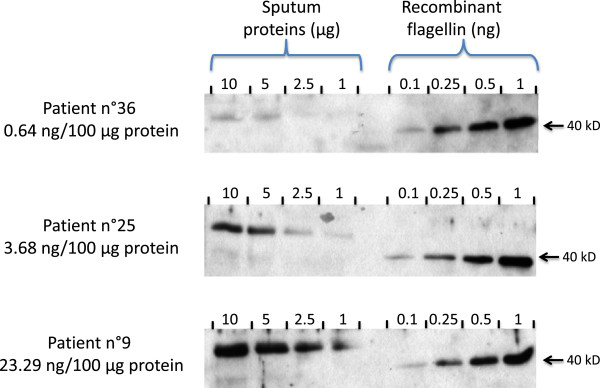
**Representative western blots of three sputum extracts containing low (n°36), medium (n°25) and high (n°9) flagellin concentrations.** Four concentrations of protein (1, 2.5, 5 and 10 μg/well) from 3 different expectorations and standard curve of flagellin (0.1, 0.25, 0.5, and 1.0 ng/well) were loaded on 10% acrylamide gel and fractionated by SDS-PAGE (see Methods). Note i) that the recombinant non glycosylated flagellin (right hand side) has a molecular weight lower (40 kD) than the native flagellin (left hand side) present in the sputum (49 kD) and corresponding to flagellin type b, and 2) the presence a faint band with a 40 kD mw observed for patients 25 and 9, and corresponding to flagellin type a.

For the quantification, total protein concentrations in expectorations were measured using Pierce BCA protein assay and then solubilized with Laemmli buffer prior to electrophoresis. Four concentrations of protein (1, 2.5, 5 and 10 μg/well) from one expectoration and the standard curve of flagellin (0.1, 0.25, 0.5, and 1.0 ng/well) were loaded on a 10% acrylamide gel and fractionated by SDS-PAGE. Proteins were electrotransferred to a polyvinylidene difluoride membrane (Immobilon, Millipore Corp., Bedford, MA) and probed using specific antibody against Pseudomonas flagellin which recognizes the two different types, flagellins a and b of *Pa* which share large stretches amino acid sequences [29]. Bound antibody was detected using the ECL + immunoblotting detection system (Thermofisher, Rockford, USA) according to the manufacturer’s instructions. Molecular masses were estimated from calibration standards included in each gel.Band intensity was analyzed by using ImageJ software version 1.45 g. A standard curve of purified flagellin was constructed using a linear curve-fit by plotting the band intensity for each standard on the y-axis against the concentration on the x-axis. Flagellin concentrations in expectorations were calculated using the equation of the standard curve. This calculation was made for each of the three or four concentrations of proteins, and the values were averaged. To normalize results, flagellin concentrations were expressed for 100 μg of total proteins. Representative immunoblots are shown in Figure 1.

### Interleukin-8 determination

IL-8 concentrations in expectorations were determined using Duo-Set ELISA kit (R&D systems, Minneapolis, USA).

### Statistical analysis

The analysis of variance (ANOVA) was performed for multiple comparisons with Bonferroni post test. For comparison of two samples Student’s *t* test was applied. A p value <0.05 was considered significant. GraphPad Prism 5 (GraphPad Software Inc, San Diego, CA) was used for the analysis.

## Results

### Patient demographics, clinical status and flagellin concentrations

The clinical characteristics of the patients and results of flagellin concentrations in sputum are shown in Table 1. Patients were classified in two groups of respiratory insufficiency according to their FEV1: severe respiratory insufficiency below 45% pred. and moderate respiratory insufficiency above 45% pred.

**Table 1:** **Patients with severe *vs *** **moderate clinical severity**

**N°**	**Sex**	**FEV1**	**FVC**	**PI/PS**	**CFRD**	**BMI**	**IL-8**	**Flagellin**
		**(% pred.)**	**(% pred.)**			**kg/m2**	**(ng/100 μg prot)**	**(ng /100 μg protein)**
**Patients with severe respiratory insufficiency**								
2	F	30	38	PI	N	15.04	2189.72	11.23
3	M	24	55	PI	Y	24.77	3163.50	5.76
8	M	44	43	PI	N	20.01	3064.52	2.92
9	M	20	39	PI	Y	20.68	1866.86	23.29
10	M	40	62	PI	Y	16.61	4009.74	33.78
13	M	44	60	PI	N	19.35	5851.31	8.58
14	M	37	69	PS	N	20.43	966.36	31.53
15	M	30	46	PI	N	18.94	7007.48	13.40
16	F	27	37	PI	N	21.22	10848.53	60.65
17	M	29	57	PI	N	22.40	2754.30	16.75
20	M	43	58	PI	N	21.26	2203.66	4.24
21	F	43	58	PI	N	20.50	3596.65	6.50
25	F	42	60	PI	Y	33.20	4089.03	3.68
31	M	38	58	PI	N	21.41	2681.30	7.17
34	F	18	22	PI	Y	21.68	3050.02	1.74
35	F	41	54	PS	N	19.20	1735.26	6.12
36	F	41	61	PI	N	18.26	3764.78	0.64
**Mean**	**M: 58.8%**	**34.76**	**51.59**	**PI: 88.2%**	**CFRD: 29.4%**	**20.88**	**3696.65**	**14.00**
±SD		8.78	12.11			3.87	2361.02	15.60
**Patients with moderate respiratory insufficiency**								
4	F	66	81	PI	N	19.89	1114.53	12.66
5	F	66	79	PI	Y	25.33	1680.15	4.80
6	F	64	73	PS	N	26.67	2748.14	5.10
7	M	60	68	PI	N	22.59	2548.47	5.62
11	F	91	106	PI	N	21.64	1832.33	3.41
12	M	59	79	PI	Y	25.69	7971.28	2.68
18	F	70	84	PI	N	25.81	2289.97	3.62
19	F	73	82	PI	Y	17.75	2142.72	30.01
22	M	75	108	PI	N	19.59	4286.10	3.82
23	F	55	89	PI	Y	20.26	1648.29	13.88
24	M	46	74	PI	Y	20.86	3194.50	4.50
26	M	55	55	PI	Y	23.66	3217.85	32.80
37	F	57	82	PI	Y	23.31	4453.10	1.07
38	M	76	91	PI	N	22.99	3437.08	2.73
**Mean**	**M: 42.8%**	**65.21**	**82.21**	**PI: 92.9%**	**CFRD: 50%**	**22.57**	**3040.32**	**9.05**
±SD		11.35	13.80			2.70	1725.97	10.15

Comparison of two groups of patients, a group with clinical severity defined as severe (18 patients), and a group with clinical severity defined as moderate (13 patients).

FEV1 (% pred.) and FCV (% pred.) are forced expiratory volume in one second and forced vital capacity (expressed as percentage of the predicted value), respectively. Pancreatic insufficiency (PI) *vs* pancreatic sufficiency (PS); cystic fibrosis-related diabetes (CFRD); Body mass index (BMI).

The mean age of the patients with severe respiratory insufficiency was 31.8 ± 9.2 years, it was of 31.5 ± 6.6 for those with moderate respiratory insufficiency (ns). We did not observe a clear-cut difference in the *CFTR* genotype between these two groups of patients (8/17 of the patients with severe disease (47%) were F508del homozygotes compared to 6/14 of the patients with moderate disease (43%)).

Comparisons of the two groups for the different clinical parameters such as pancreatic insufficiency (PI) *vs* pancreatic sufficiency (PS), cystic fibrosis-related diabetes (CFRD) and body mass index (BMI) did not demonstrate obvious differences as well (Table 1).

IL-8 measurements, done as a proxy for the ongoing inflammatory response also did not demonstrate a difference according to the category of respiratory insufficiency of our patients (Table 1). The concentrations ranged from 966 to 10,848 ng/100 μg proteins.

Finally, for the parameter constituting the aim of our study, *i.e.* the concentration of flagellin in the sputum, the difference was not statistically significant between the two groups, with mean values ± sd of 14.0 ± 15.6 ng/100 μg proteins for patients with severe respiratory insufficiency and of 9.1 ± 10.1 ng/100 μg proteins (Table 1).

### Correlation of flagellin concentrations in sputum with respiratory insufficiency

In order to visualize differently a possible correlation between flagellin concentrations and patient respiratory insufficiency, we considered two groups, *i.e.* a group with flagellin concentrations lower than 10 ng/100 μg proteins (20 patients), and a group with flagellin concentrations higher than 10 ng/100 μg proteins (11 patients). The mean ± sd values for flagellin were thus 4.2 ± 2.0 and 25.5 ± 14.7 ng/100 μg proteins, respectively (Table 2).

**Table 2:** **Patients with high *vs *** **low flagellin concentrations**

**N°**	**Sex**	**Status**	**Mucoid/non mucoid**	**FEV1**	**FVC**	**Total proteins**	**IL-8**	**Flagellin**
		**Stable/Exacerbation**		**(% pred.)**	**(% pred.)**	**(mg/ml)**	**(ng/100 μg prot)**	**(ng/100 μg protein)**
**Patients with flagellin sputum < 10 ng/100 μg protein**								
3	M	Stable	Non mucoid	24	55	6.58	3163.50	5.76
5	F	Stable	Non mucoid	66	79	3.21	1680.15	4.80
6	F	Exacerbation	Mucoid	64	73	4.57	2748.14	5.10
7	M	Stable	Non mucoid	60	68	9.34	2548.47	5.62
8	M	Exacerbation	Non mucoid	44	43	3.84	3064.52	2.92
11	F	Stable	Non mucoid	91	106	3.15	1832.33	3.41
12	M	Stable	Non mucoid	59	79	4.39	7971.28	2.68
13	M	Stable	Non mucoid	44	60	11.8	5851.31	8.58
18	F	Stable	Non mucoid	70	84	4.17	2289.97	3.62
20	M	Stable	Non mucoid	43	58	8.68	2203.66	4.24
21	F	Stable	Mucoid	43	58	5.91	3596.65	6.50
22	M	Stable	Non mucoid	75	108	5.87	4286.10	3.82
24	M	Stable	Non mucoid	46	74	7.25	3194.50	4.50
25	F	Stable	Non mucoid	42	60	4.91	4089.03	3.68
31	M	Stable	Mucoid	38	58	4.70	2681.30	7.17
34	F	Exacerbation	Non mucoid	18	22	4.22	3050.02	1.74
35	F	Stable	Non mucoid	41	54	7.83	1735.26	6.12
36	F	Exacerbation	Non mucoid	41	61	6.71	3764.78	0.64
37	F	Stable	Mucoid	57	82	8.93	4453.10	1.07
38	M	Stable	Non mucoid	76	91	6.59	3437.08	2.73
**Mean**	**M: 50%**		**non.mucoid:.80%**	**52.10**	**68.65**	**6.13**	**3382.06**	**4.24**
±SD				18.11	20.39	2.30	1491.93	2.03
**Patients with flagellin sputum > 10 ng/100 μg protein**								
2	F	Stable	Mucoid	30	38	7.90	2189.72	11.23
4	F	Stable	Non mucoid	66	81	3.25	1114.53	12.66
9	M	Stable	Mucoid	20	39	8.22	1866.86	23.29
10	M	Stable	Mucoid	40	62	4.11	4009.74	33.78
14	M	Stable	Non mucoid	37	69	5.38	966.36	31.53
15	M	Stable	Non mucoid	30	46	6.42	7007.48	13.40
16	F	Exacerbation	Non mucoid	27	37	5.87	10848.53	60.65
17	M	Stable	Non mucoid	29	57	2.79	2754.30	16.75
19	F	Stable	Non mucoid	73	82	5.14	2142.72	30.01
23	F	Stable	Mucoid	55	89	6.81	1648.29	13.88
26	M	Stable	Non mucoid	55	55	8.44	3217.85	32.80
**Mean**	**M: 54.5%**		**Non mucoid:.63.6%**	**42.00**	**59.55**	5.85	**3433.31**	**25.45**
±SD				17.52	18.80	1.94	2981.86	14.65

Comparison of two groups of patients, a group with flagellin concentrations lower than 10 ng/100 μg proteins (20 patients), and a group with flagellin concentrations higher than 10 ng/100 μg proteins (11 patients).

FEV1 (% pred.) and FCV (% pred.) are forced expiratory volume in one second and forced vital capacity (expressed as percentage of the predicted value), respectively.

For the group of patients with low flagellin concentrations the mean age was 31.1 ± 6.8 years, with a 50/50 male/female ratio, and for the group of patients with high flagellin concentrations the mean age was 32.6 ± 10.1 years, with 54/46 male/female ratio. Means ± sd values were not statistically significant.

In fact for FEV1 there was no significant differences as well, values being of 52.1 ± 18.1% of predicted and 42.0 ± 17.5% (means ± sd) of predicted for the low and high flagellin concentration groups, respectively. Thus, higher flagellin concentrations did not automatically suggest more severe disease.

We also assessed whether high concentrations of flagellin may be linked with pulmonary exacerbations *i.e.* acute worsening of CF symptoms caused by infection and that lead to the need for additional antibiotic treatment [7]. Five patients had mild pulmonary exacerbation requiring an antibiotic IV course at home, none was hospitalized. No correlation was observed as patients with exacerbation had very different concentrations of flagellin in their expectoration, ranging from 0.64 to 60.65 ng/100 μg proteins, in fact the two extreme values of the whole group of 31 patients. Among the 20 patients with flagellin concentrations lower than 10 ng/100 μg proteins, 4 patients had exacerbations, compared to only one patient among the 11 other patients whose flagellin concentrations were higher. Thus, there was no correlation between flagellin concentrations and exacerbations.

Analysis of the expectorations did not demonstrate differences among the total proteins in sputum between the two groups (Table 2). As well, no correlation (r^2^ = 0.02) could be found between flagellin concentrations and bacterial numbers.

Interestingly, mucoid strains were present in 4/20 patients (20%) with low flagellin concentrations and even more in 4/11 (36%) patients with high flagellin concentrations. As discussed previously the general statement is made that mucoid strains lose the ability to make flagellin, however regardless of whether the patient carried or not a mucoid strain of *Pa*, the mean concentration of flagellin found in the sputum samples was similar, *i.e.* 13.6 ± 11.4 *vs* 12.5 ± 15.2 ng/100 μg, respectively. It should however be noted that the clinical laboratory reported the presence or not of a mucoid strain which does not imply that when this phenotype was present, the nonmucoid strain was absent.

## Discussion

Flagellin is a potent activator of a broad range of cell types involved in innate and adaptive immunity. Its recognition by TLR5 is involved in activating pulmonary defenses against *Pa* that leads eventually to elimination of this bacterium from the host. Thus, bacterial mutants that lacked flagellin appeared to evade immune control and were relatively slowly cleared from the lungs. Along the same line, the loss of the TLR5 response has consequences on the host response, that results in an impairment of antimicrobial effectors [30].

The well known paradox is that the TLR5-flagellin interaction is a major mediator of inflammation following exposure to *Pa*. Indeed, *Pa* mutants which overproduced flagellin, caused severe inflammation [31]. The consequence is that some authors have proposed TLR5 as an anti-inflammatory target [32]. Interestingly, CF patients carrying the TLR5 premature stop codon had a higher body mass index than CF patients homozygous for the functional allele. This is some evidence that a loss of TLR5 function resulting in reduced flagellin responsiveness is associated with improved health indicators in adults with CF. Unfortunately, improvements in lung functions were not statistically significant [32]. Another study demonstrates that flagellin induces the generation of myeloid-derived suppressor cells and suggest that *Pa* uses this mechanism to undermine T cell–mediated host defense in CF [33]. In summary, there is not a clear picture about the beneficial or deleterious consequences of TLR5-flagellin interaction *in vivo*. And in fact, it was even not known whether flagellin was present or not in the sputa of CF patients.

## Conclusion

The present study although having a small sample size, allows two conclusions. Firstly, it points out that possibly all *Pa* colonized patients, regardless of whether the clinical laboratory indicates they are colonized with mucoid strains, carry clones of *Pa* that express flagellin. This is the first time that flagellin concentrations in the sputum of CF patients has been examined. Thus caution should be exercised in concluding that patients with mucoid strains cease to carry flagellin producing clones. This finding is consistent with a recent report that there is significant “phenotypic” heterogeneity of *Pa* populations in a CF patient [34]. Secondly, while flagellin seems to be always present in CF airways, it is difficult to measure its contribution to inflammation (IL-8 measurements) and lung function deterioration, since we could not correlate amounts present with the level of respiratory insufficiency nor with the presence of a pulmonary exacerbation. However given that this agonist has now been shown to be present in all patients we studied, attempts to down regulate inflammation by the use of TLR5 antagonists still remain viable.

Although we are aware that the present data are not conclusive and need to be strenghtened, we believe that their novelty (this is the first demonstration that flagellin is present in the sputum of all patients studied) deserves to be brought to the attention of the CF community.

## Competing interests

The authors declare that they have no competing interests.

## Authors’ contributions

VB carried out the laboratory experiments (protein measurements, western blots, immunoassays) and performed the statistical analysis. GT carried out the laboratory experiments (preparation of the expectorations). TB analyzed and provided patient genotypes. PM analyzed and provided patient microbiological data. HC and AC participated in the study design. RR participated in the design of the manuscript and helped to draft it. VB, DH and MC conceived of the study, and participated in its design and coordination and helped to draft the manuscript. All authors read and approved the final manuscript.

## Pre-publication history

The pre-publication history for this paper can be accessed here:

http://www.biomedcentral.com/1471-2466/14/100/prepub
